# Unsupervised and quantitative intestinal ischemia detection using conditional adversarial network in multimodal optical imaging

**DOI:** 10.1117/1.JMI.9.6.064502

**Published:** 2022-11-28

**Authors:** Yaning Wang, Laura Tiusaba, Shimon Jacobs, Michele Saruwatari, Bo Ning, Marc Levitt, Anthony D. Sandler, So-Hyun Nam, Jin U. Kang, Jaepyeong Cha

**Affiliations:** a Johns Hopkins University, Department of Electrical and Computer Engineering, Baltimore, Maryland, United States; b Children’s National Hospital, Division of Colorectal and Pelvic Reconstruction, Washington, District of Columbia, United States; c Children’s National Hospital, Sheikh Zayed Surgical Institute, Washington, District of Columbia, United States; d Dong-A University Medical Center, Department of Surgery, Busan, Republic of Korea; e George Washington University School of Medicine and Health Sciences, Department of Pediatrics, Washington, District of Columbia, United States

**Keywords:** laser speckle contrast imaging, deep learning, unsupervised anomaly detection, generative adversarial network, multimodal optical imaging, dye-free tissue perfusion assessment

## Abstract

**Purpose:**

Intraoperative evaluation of bowel perfusion is currently dependent upon subjective assessment. Thus, quantitative and objective methods of bowel viability in intestinal anastomosis are scarce. To address this clinical need, a conditional adversarial network is used to analyze the data from laser speckle contrast imaging (LSCI) paired with a visible-light camera to identify abnormal tissue perfusion regions.

**Approach:**

Our vision platform was based on a dual-modality bench-top imaging system with red-green-blue (RGB) and dye-free LSCI channels. Swine model studies were conducted to collect data on bowel mesenteric vascular structures with normal/abnormal microvascular perfusion to construct the control or experimental group. Subsequently, a deep-learning model based on a conditional generative adversarial network (cGAN) was utilized to perform dual-modality image alignment and learn the distribution of normal datasets for training. Thereafter, abnormal datasets were fed into the predictive model for testing. Ischemic bowel regions could be detected by monitoring the erroneous reconstruction from the latent space. The main advantage is that it is unsupervised and does not require subjective manual annotations. Compared with the conventional qualitative LSCI technique, it provides well-defined segmentation results for different levels of ischemia.

**Results:**

We demonstrated that our model could accurately segment the ischemic intestine images, with a Dice coefficient and accuracy of 90.77% and 93.06%, respectively, in 2560 RGB/LSCI image pairs. The ground truth was labeled by multiple and independent estimations, combining the surgeons’ annotations with fastest gradient descent in suspicious areas of vascular images. The total processing time was 0.05 s for an image size of 256×256.

**Conclusions:**

The proposed cGAN can provide pixel-wise and dye-free quantitative analysis of intestinal perfusion, which is an ideal supplement to the traditional LSCI technique. It has potential to help surgeons increase the accuracy of intraoperative diagnosis and improve clinical outcomes of mesenteric ischemia and other gastrointestinal surgeries.

## 1 Introduction

Bowel ischemia is a life-threatening medical condition caused by decreased or blocked blood flow to the intestine.^1^ It is associated with a wide range of gastrointestinal diseases, such as intestinal volvulus, obstruction, various colonic pull-throughs, or abdominal neoplasms.^2^ In acute presentations sudden arterial or venous insufficiency can lead to a surgical emergency, known as acute intestinal ischemia,^3^ which accounts for 0.1% of acute hospital admissions in the United States and Europe^4^^,^^5^ with an overall mortality rate of ∼70%.^3^ If left untreated, it rapidly progresses to irreversible intestinal necrosis, leading to fatal metabolic disorders and end-organ dysfunction.^6^^,^^7^ Therefore, timely surgical treatment is essential for re-establishing the intestinal blood flow. During a variety of operative treatment, surgeons must routinely and precisely assess bowel viability to determine the resection margin to preserve as much of the viable bowel as possible.^8^ Moreover, if the non-viable bowel is not identified, it may lead to bowel necrosis, sepsis, shock, or a potentially fatal clinical emergency, whereas removing viable bowel unnecessarily can lead to short bowel syndrome.^9^ Owing to the lack of reliable markers, assessment of bowel ischemia in clinical practice is subjective and depends on the individual surgeon’s experience level, and their subjective visual inspection. Bowel ischemia is manifested mainly through the color of the serosa, bowel peristalsis, and bleeding from the arteries.^10^ Therefore, a more quantitative and objective measurement of intestinal perfusion is needed. Several intraoperative techniques have been used to evaluate intestinal viability, including laser Doppler flowmetry, visible-light spectrophotometry, and laser fluorescence angiography. However, laser-Doppler flowmetry has a very limited measurement area and is also a contact measurement method that may affect the local blood flow.^11^ Fluorescence angiography requires intravenous dye injection, such as indocyanine green (ICG). It can cause allergic reaction and is only available for short time ranges after the injection.^12^

More recently, laser speckle contrast imaging (LSCI) has been introduced to gastrointestinal surgeries to visualize tissue microcirculation in real time.^13^^–^^16^ Unlike laser Doppler flowmetry and ICG imaging, LSCI is noncontact and agent-free. The movement of red blood cells can be measured by analyzing the speckle patterns generated by coherent monochromatic laser light.^17^ The complicated scattering process makes LSCI a semi-quantitative technique requires a fine-tuned calibration procedure. Some researchers address this by fixing a point on the scale and considering it a perfusion unit.^18^ However, no comprehensive solution exists for enabling inter-patient comparability, which impedes its clinical applications.^19^ For this purpose, surgeons must create a zero-flow area without perfusion or motion, which is impractical during surgery.^19^ Although several pilot studies have concluded that different critical values impede tissue healing^20^^,^^21^ within 25% or 40% of their defined baselines, these results cannot be generalized. Therefore, accurate identification of abnormal intestinal perfusion in real time based on LSCI remains difficult for surgeons with little experience.

In recent years, machine learning, in particular unsupervised learning,^22^ has been reported to be useful in computer-aided anomaly detection in brain imaging. It refers to perform learning tasks without the guidance of labeled ground truth. One key idea is to learn the distribution of the normal anatomy during training and highlight the diseased regions during testing by comparing the input with the learned healthy representations.^23^ Some initial work has been completed on an autoencoder that can compress high-dimensional data into lower-dimensional encoder representation.^24^^,^^25^ Leveraging this capability, abnormal results can be obtained in unseen diseased regions, which is helpful for distinguishing diseased regions from healthy data. The study achieved a Dice coefficient of 0.55 on the brain magnetic resonance imaging (MRI) dataset.^23^ More recently, the generative adversarial network (GAN),^26^ proposed by Goodfellow et al., has received increasing attention, as it can generate new and realistic-looking images from learned distributions. Inspired by the strong generative power of GANs, this study presents a new surgical vision platform that utilizes unsupervised deep learning algorithms to generate a quantitative map for predicting the degree of intestinal ischemia. Our proposed method is based on a benchtop dual-modality imaging system with color red-green-blue (RGB) and LSCI channels, as previously prototyped by our group.^15^^,^^27^^,^^28^ First, to mitigate the inherent misalignment issues of multimodality optical systems, a conditional GAN (cGAN)-based multimodal image registration network was designed. Second, the processed image pairs were trained and tested using another cGAN-based predictive model. The predicted data were compared with the data originally obtained from LSCI. The resulting probability map was used to differentiate non-viable/viable intestinal tissues using quantification values. Our method was evaluated based on open surgeries conducted on live swine, including vascular clamping and intestinal anastomosis tests.

## 2 Method

### 2.1 Overview

**Fig. 1:**
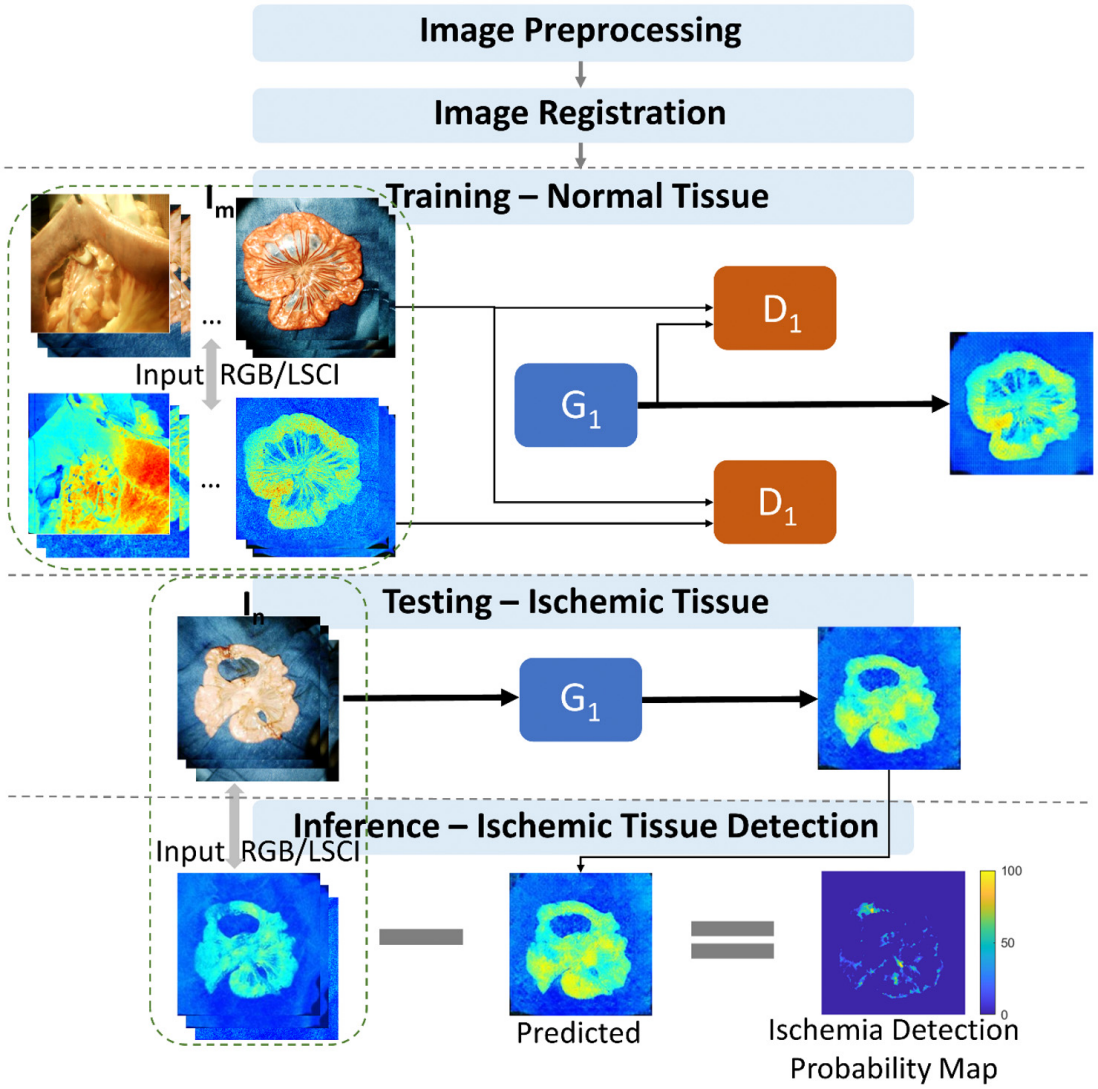
Proposed pipeline for detecting ischemic bowel tissue using RGB/LSCI images with cGAN.

Figure 1 shows the framework for predicting normal and abnormal tissue perfusion in RGB and LSCI images. First, dual-channel images were captured using our imaging system and preprocessed through normalization and histogram equalization. All image pairs containing the diseased bowels were carefully cropped to exclude any possible ischemic regions. An unsupervised registration method based on cGAN was adopted to achieve a precise alignment of the multimodality dataset. The well-aligned images were split into two groups for the following generative model:

The training dataset $I_{m}$ comprised normal patterns. The uncropped version of the abnormal pattern $I_{n}$ was used for testing. The difference between the learned norm and original LSCI was measured. After post-processing, this similarity map provided a quantitative description of the blood flow level. Model evaluation was performed through a comparison with manual annotation by experienced surgeons.

### 2.2 System Setup

**Fig. 2:**
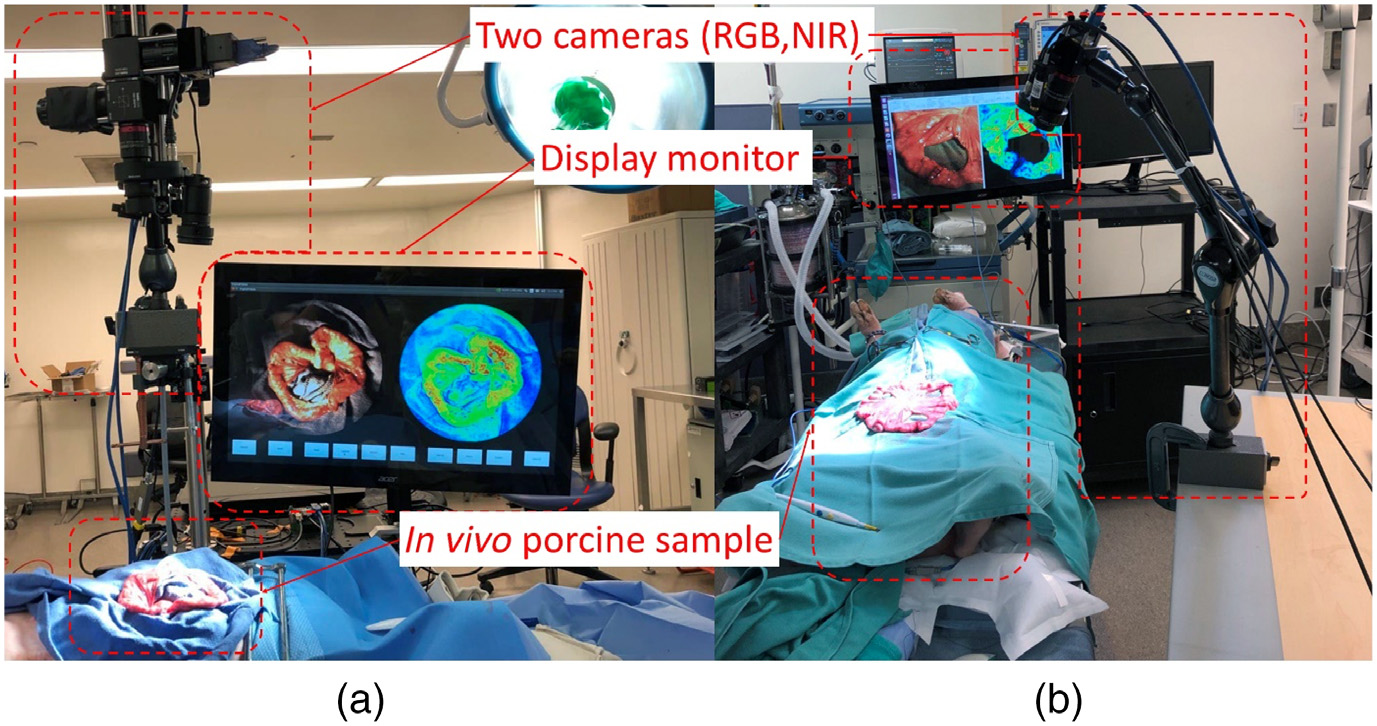
(a) and (b) Examples of the *in vivo* porcine small bowel studies.

Figure 2 shows the schematic of the system setup. Visible-light and near-infrared (NIR) cameras shared a common optical path separated by a beam splitter such as to simultaneously obtain standard color images and LSCI images. The data acquisition rates were 30 Hz for the visible-light camera and 70 Hz for the NIR camera. Further details can be found in previous publications from our group.^15^^,^^27^^,^^28^

Laser speckle is a type of interference pattern from scattered or reflected light when illuminating a random medium and is roughly defined as follows:

$$K=\frac{\sigma }{\left\langle I\right\rangle },$$ (1)

where the speckle contrast K is the ratio of the standard deviation of the intensity σ to the average intensity ⟨I⟩ over the window. Here, the inverse relationship between the flow velocity and the square of the speckle contrast K is used, as verified in Refs. 29 and 30, to describe the change in local blood flow. Depending on the application, the speckle contrast K can be calculated using the temporal integral, spatial integral, or both. To minimize the background artifacts from the physiological motion of the experiment subjects and ensure an adequate sampling rate, a 5×5 spatial window size was utilized.

### 2.3 Data Acquisition and Image Preprocessing

To evaluate the performance of our model, four animal studies were conducted on swine (protocol #30591 at the Children’s National Hospital). To induce ischemia, two surgical interventions were performed. Surgeons simply placed clamps on corresponding regions for bowel resection and subsequent anastomosis on *in vivo* porcine samples. They marked the maximum possible areas with abnormal tissue perfusion based on the surface color and bowel peristalsis. A total of 35 datasets were collected using the dual-channel benchtop imaging system, of which 15 contained healthy tissue images used for training and the remaining 20 contained abnormal tissue perfusion region images used for testing. All 35 image sequences were split into two groups without any overlaps. None of the cases in the training or testing datasets focused on similar intestinal regions during a similar period. To ensure the diversity of our datasets, six experimental conditions were implemented during data acquisition, including distinct regions of the small bowels, ischemic levels, ischemic time, reperfusion injury, image background, camera angles, and lighting conditions. Surgical towels were used in three animal studies for a clean background, and the remaining study was conducted inside the abdomen for obtaining healthy samples. Each dataset consisted of 128 RGB and LSCI images. The image size was 1024×1024  pixels. Over each recording period (4.27 s, 128 frames, and 30 FPS), RGB images appeared similar except for small organ motions; however, the mean intensity of LSCI images tended to fluctuate up to 10% because of the motion induced by respiration, cardiac pulsation, and intestinal peristalsis. This variation is uneven and follows multiple frequencies; therefore, it is crucial to evaluate the performance of our model under these physiological motions.

**Fig. 3:**
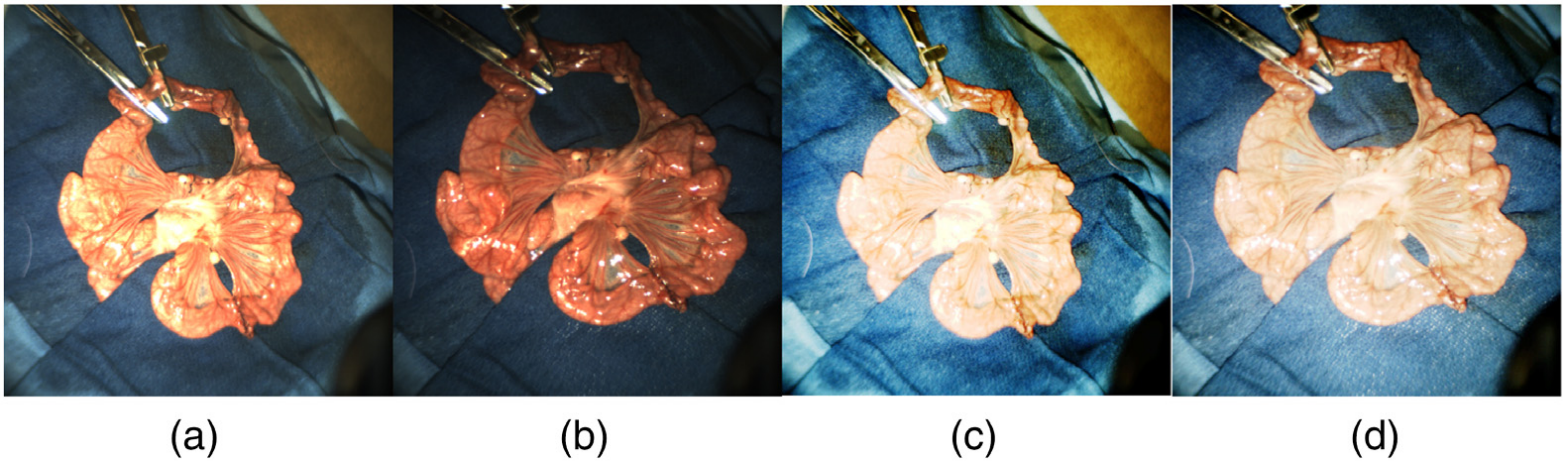
Examples of *in vivo* small bowel RGB images of swine (a) and (b) before and (c) and (d) after histogram equalization under different illumination conditions.

Appropriate degrees of down sampling and averaging were applied to the raw datasets, which could decrease data redundancy while maintaining all the details related to the blood flow changes associated with ischemia or reperfusion, which vary over time. Limited by the receptive field and network depth, images had to be down sampled twice (256×256) before being fed into the model. All images were normalized to the [−1,1] range, which can accelerate the learning process and the convergence of the cost function. The different illumination conditions, camera angle, and movement of the imaging device can cause under or overexposure of images. Here, a simple histogram equalization method was applied to standard color images to increase the global contrast and extract more details, as shown in Fig. 3.

To illustrate that the training and testing data were independent, 100 representative samples from each group were selected. The average mean square error between the training and testing LSCI images was 0.6091, with a standard deviation of 0.2879. It is worth noting that no additional pre-trained datasets were used, and no transfer learning was applied in our experiments. No other data augmentation methods were used.

### 2.4 Unsupervised Learning for Image Registration

**Fig. 4:**
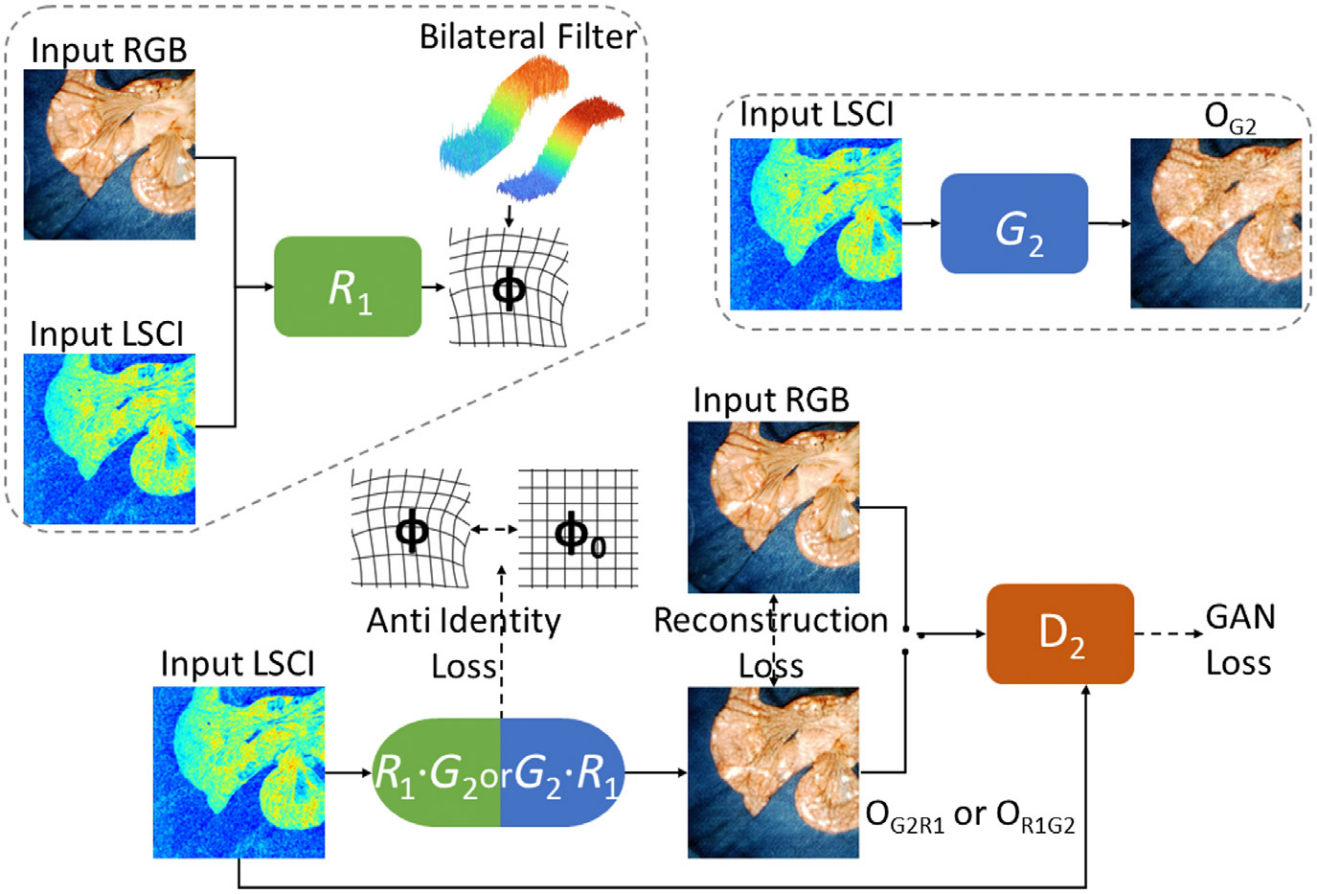
Proposed pipeline for unsupervised multimodal image registration using RGB/LSCI datasets with cGAN and STN.

A misalignment can exist between RGB and LSCI images because of a discrepancy in lens parameters, light spectrum, and relative positions, hampering the ability to combine cross-modality features. Compared with color images, speckle images lack parts of texture information, and only microcirculation is emphasized. Recently, Arar et al.^31^ designed a network for unsupervised multimodality image registration, which was first employed on RGB and NIR images. Inspired by this study, we modified their model to fit our dataset, as shown in Fig. 4. The model consists of three components: generator $G_{2}$, discriminator $D_{2}$, and a spatial transformation network (STN)^32^ $R_{1}$ similar to a GAN.^26^ The generator $G_{2}$ is implemented to transfer an input image to another domain with the same content representation, specifically, to map the vascular images to standard-color images. The specific architecture contains repeated stacks of down/up sampling layers, as given in Table 1. During eight down/up samplings, convolutional/deconvolutional layers were applied to the quarter/quadruple activation areas. Some of the common settings in GANs were employed, such as the leaky rectifier linear unit for each block of the encoder and a hyperbolic tangent (Tanh) activation function in the last layer.^33^ Skip connections were used to maintain low-level information against bottlenecks, where the batch size is the smallest. Compared to the generator, discriminator $D_{2}$ is simpler. $D_{2}$ is a common classification model trained to identify real RGB images from fake images (generated). It uses convolutional blocks to extract different feature levels and returns a probability matrix. The registration network $R_{1}$ aims to generate a non-rigid deformation field to predict the spatial alignments for specific LSCI images based on the thin-plate spline transformation.^34^ The network has a large degree of freedom in describing the pixelwise two-dimensional motion. The entire information flow first passes through $R_{1}$ and then $G_{2}$, or vice versa, simultaneously. This commutative property ensures that there is no functional interference with each other.

**Table 1:** Architectural details.

	Network architectures (feature map)	Number of parameters (in millions)	Kernel size of convolution window	Input image size
$G_{1}$	3→64→128→256→512→1024→512→256→128→3	54.425	4	[256,256,3]
$D_{1}$	6→64→128→256→512→1	2.770	4	[256,256,6]
$G_{2}$	3→64→128→256→512→1024→512→256→128→3	54.410	4	[256,256,3]
$D_{2}$	6→64→128→256→512→1	2.768	4	[256,256,6]
$R_{1}$	6→32→64→128→64→32→2	2.059	3	[256,256,6]

The dashed lines with arrows in Fig. 4 show the objective of our network, which can be specified as

$$L_{\mathrm{cGAN}}(G_{2},D_{2},R_{1})=E[\log(D_{2}(I_{\mathrm{RGB}},I_{\mathrm{LSCI}}))]+E[\log(1-D_{2}(O_{R_{1}G_{2}},I_{\mathrm{LSCI}}))]\;+E[\log(1-D_{2}(O_{G_{2}R_{1}},I_{\mathrm{LSCI}}))],$$ (2)

$$L_{\text{smooth}}(\phi ,v)=\underset{u\in N(v)}{\sum }B(u,v)\|\phi (u)-\phi (v)\|,$$ (3)

$$L_{\mathrm{recon}}(G_{2},R_{1})=E[{\left\|O_{R_{1}G_{2}}-I_{\mathrm{RGB}}\right\|}_{1}]+E[{\left\|O_{G_{2}R_{1}}-I_{\mathrm{RGB}}\right\|}_{1}],$$ (4)

$$L_{\mathrm{anti}}(G_{2},R_{1})=E[{\left\|O_{G_{2}}-I_{\mathrm{RGB}}\right\|}_{1}]+E[{\left\|O_{R_{1}}-I_{\mathrm{LSCI}}\right\|}_{1}],$$ (5)

$$L_{\text{total}}=\arg\;\underset{R,G_{2}}{\min}\;\underset{D_{2}}{\max}\;L_{\mathrm{cGAN}}(G_{2},D_{2},R_{1})+\lambda_{R}L_{\mathrm{recon}}(G_{2},R_{1})+\lambda_{S}L_{\text{smooth}}(R_{1})+\lambda_{A}L_{\mathrm{anti}}(G_{2},R_{1}).$$ (6)

Here, the deformation ϕ(v)=(Δy,Δx) is at pixel v=(i,j), B(u,v) denotes a bilateral filter,^31^ and N(v) is a 3×3  pixel neighborhood. $L_{\text{smooth}}$ encourages a similar deformation ϕ within neighboring pixels without an acnode in any 3×3 neighborhood. In other words, it measures the smoothness and continuity of deformation fields and prevents interior fractures. $L_{\mathrm{recon}}(G_{2},R_{1})$ stated in Eq. 4 measures the absolute errors of each pixel even outside the boundaries, which encourages $G_{2}$ to create realistic-looking images and maintain the original intestinal geometric structures. $O_{\left[X\right]}$ refers to the output from a certain network X and $E_{\left[X\right]}$ refers to the expected value of a certain variable X. Our experience is that a large $\lambda_{S}$ prevents the movement of the target images. In practice, unprocessed misalignment errors are observed when there are few input images, ∼40, with a pixel size of 1024×1024. Under these circumstances, the network architecture was modified by adding $L_{\mathrm{anti}}$ to prevent premature convergence, as shown in Eq. 5. Specifically, a penalty term $L_{\mathrm{anti}}$ was defined on the identity transformation field. Without it, $G_{2}$ may generate realistic-looking images and spatial transformations simultaneously while bypassing $R_{1}$. In practice, we set $\lambda_{R}=150$, $\lambda_{S}=130$, and $\lambda_{A}=-2.0$. Early stopping was used to reduce overfitting by monitoring the performance of certain epochs.

### 2.5 Unsupervised Learning for Anomaly Detection

#### 2.5.1 Training a model of healthy anatomy

The key idea behind our method is to train a generative model of healthy anatomy using unsupervised learning. Validated by Goodfellow et al.^26^ and Radford et al.,^35^ GAN has a strong ability for generating realistic new samples following the same statistics as those of the training set. In light of this, Schlegl et al.^36^ presented the first anomaly detection technique based on GAN, named AnoGAN, which was implemented for optical coherence tomography images of the retina. However, our objective is cross-modality information fusion, and simply transfer learning from AnoGAN is not technically feasible. Therefore, a cGAN-based^37^ model was utilized for normal/ischemia prediction. As a variation of the standard GAN, it has an extra input layer for additional data from various sources, which is particularly suitable for image-to-image translation tasks.^38^ As shown in Fig. 1, an adversarial generative model was trained on a preprocessed set of medical images $I_{m}\in R^{a\times b}$, and tested on $I_{n}\in R^{a\times b}$. The network input was RGB/LSCI image pairs. The output was reconstructed from the LSCI images. Our model was trained to learn the correspondence between the morphological features and normal blood perfusion levels. Here, generator $G_{1}$ is in the opposite direction of $G_{2}$. Discriminator $D_{1}$ also performs the same task as $D_{2}$, which consists of identifying real images from generated ones in the form of probability. The network design for $G_{1}$ and $D_{1}$ is also similar to $G_{2}$ and $D_{2}$ with minor changes in collaborating with STN, as detailed in Table 1. To avoid blurring problems,^39^ a convolutional PatchGAN classifier^38^ was utilized for both $D_{1}$ and $D_{2}$, such that the final output was a probability matrix for different image patches. The network objective can be stated as

$$L_{\mathrm{cGAN}}(G_{1},D_{1})=E[\log(D_{1}(I_{\mathrm{RGB}},I_{\mathrm{LCSI}}))]+E[\log(1-D_{1}(I_{\mathrm{RGB}},O_{G_{1}}))],$$ (7)

$$L_{\mathrm{recon}}(G_{1})=E[{\left\|O_{G_{1}}-I_{LCSI}\right\|}_{1}],$$ (8)

$$L_{\text{total}}=\arg\;\underset{G_{1}}{\min}\;\underset{D_{1}}{\max}\;L_{\mathrm{cGAN}}(G_{1},D_{1})+\lambda L_{\mathrm{recon}}(G_{1}).$$ (9)

$L_{\mathrm{recon}}(G_{1})$ measures the distance between the synthesized and ground truth images. It penalizes outputs that are irrelevant to the RGB/LSCI image pairs. As a result, it alleviates the mode collapse issue,^40^ which is a common problem faced by GAN. Unlike Eq. 6, the contribution of spatial alignment ($L_{\text{smooth}}$) is excluded from Eq. 9. Compared to other common loss functions in machine learning, such as the mean square error ($L_{2}$) loss, the goal of $L_{\mathrm{cGAN}}$ is not to measure a certain type of error in predictions, but to prompt $G_{1}$ and $D_{1}$ to work competitively. $G_{1}$ attempts to minimize $L_{\mathrm{cGAN}}$, whereas $D_{1}$ attempts to maximize it. $L_{\text{total}}$ represents the final objective. In practice, λ=100. GAN models often suffer from several challenges, such as non-convergence. If left unsolved, no realistic-looking speckle images are predicted, leading to high potential abnormalities. To stabilize the training procedure, batch normalization layers are applied to our design, similar to those depicted in Ref. 35. They standardize the layer inputs and transition the flow of gradients into a deeper structure. We also implemented a solution of adopting a dropout at 50% rate in $G_{1}$ for this scenario. This reduces the dependence between neurons and avoids overfitting.

#### 2.5.2 Detection of anomalies

In this section, the discrepancy between real images and their reconstructed representations in pathological regions is analyzed. The preprocessing and image registration steps ensure that no environmental factors influence the outcomes. The similarity (or difference) measurement is given as

r=|y−y^|, (10)

where y is the LSCI image of the test sample; y^ is the reconstructed LSCI image from cGAN; and r is the corresponding residual. To exclude background noise, the tissue is roughly separated from the background through a binarization of RGB images^41^ and further multiplied by matrix r. Moreover, r is processed by morphological closing to remove small holes and maintain internal connectivity. Matrix r can provide a discriminative score for poorly (or well) perfused regions of the small bowels. The core concept behind this is that there are only small morphological differences between RGB images of normal and abnormal tissues. Mapping of the encoded latent representation to abnormal speckle patches is not learned during training. As a result, the generator $G_{1}$ attempts to translate the abnormal RGB patches into normal speckle patches, leading to erroneous recovery results.

### 2.6 Implementation Details

The pipelines for cGAN-based image registration and pathological detection were both written in Python under the Google Colab platform equipped with a high graphics processing unit random access memory (25 GB). Manual image annotations and quantitative evaluations were implemented in MATLAB. The entire training cycle for image alignment, training, and inference took 6 h. The learning rate was set to 0.0002 for $G_{1}$ and $D_{1}$, and 0.001 for $G_{2}$, $D_{2}$, and $R_{2}$, both with the Adam optimizer^42^ with the momentum term β=0.9. The batch size was 1. After convergence of the cGAN-based model, generally within 40 epochs, abnormal tissue perfusion detection was performed by testing the model on a dataset not used for training. The entire calculation time was 0.05 s for one frame, including image-to-image registration, translation, and similarity measurement.

### 2.7 Ground Truth Definition and Evaluation Metrics

#### 2.7.1 Evaluation of multimodal image registration model

To evaluate the registration accuracy of multimodal images, 140 datasets $\left\{I_{n,}I_{m}\right\}$ were randomly selected and manually labeled. Each LSCI/RGB pair contained 7 to 9 annotation pairs. Most of these were salient landmarks. Half of the object landmarks were located on the clear outline or corner of the mesentery and bowel in red, whereas the other half denoted interior intestinal walls, stitch marks, and large mesenteric vessels in black. The remainder of the annotation pairs 0 to 2, such as occlusion clamps and surgical towels, were placed on the background of the scene in green. An example was shown in the following result section. The registration error was defined by the distance between corresponding annotation pairs. Introducing interior black annotations resulted in a better evaluation of the interior alignment.

#### 2.7.2 Evaluation of ischemia detection model

To comprehensively evaluate our predictions of ischemia areas, two different strategies were employed on defining the ground truth. First, an experienced surgeon was asked to assess the medical condition of the *in vivo* porcine bowel and label the resection margins. This is a subjective rough estimation based on visual tests.^10^ In the case of uncertainty, surgeons tend to resect all possible non-viable areas to prevent postoperative complications, which can reasonably explain why in the ground truth, this area may be larger than the delineated region of our model. It is unfair to take them as the baseline. Under these circumstances, another type of ground truth was defined to depict the variations in the blood flow. As shown in Fig. 1, smooth and gradual transition zones were observed in the LSCI images without a fixed threshold or gradient value, which prompted the design of our model. Furthermore, this created challenges in defining the ideal annotations. For better evaluation, 140 LSCI/RGB image pairs were randomly selected and regions of interest (ROIs), referred to as the transition zones, were manually set up. Thus, a contour map was employed, in which two dominant intensity levels were automatically selected by computers. For each image pair, this process was repeated three times, and the results were averaged. The averaging process further improved the reliability of the handcrafted annotations. Image examples were shown in the following result section.

The segmentation result of our ischemia detection model was binarized before comparing it with the second type of the ground truth, whereby the abnormality rate was reduced to 10%. The performance metrics included accuracy (AC), Dice coefficient (DC), sensitivity (SE), specificity (SP), and precision (PE). The following equations were used to calculate these metrics:

$$\mathrm{AC}=\frac{\mathrm{TP}+\mathrm{TN}}{\mathrm{TP}+\mathrm{TN}+\mathrm{FP}+\mathrm{FN}},\;\mathrm{DC}=\frac{2\mathrm{TP}}{2\mathrm{TP}+\mathrm{FP}+\mathrm{FN}},$$ (11)

$$\mathrm{SE}=\frac{\mathrm{TP}}{\mathrm{TP}+\mathrm{FN}}\;\mathrm{SP}=\frac{\mathrm{TN}}{\mathrm{TN}+\mathrm{FP}},\;\mathrm{PE}=\frac{\mathrm{TP}}{\mathrm{TP}+\mathrm{FP}},\;$$ (12)

where TP, TN, FP, and FN refer to true positive, true negative, false positive, and false negative, respectively. The area under the curve (AUC) was further utilized for performance assessment.

## 3 Results

### 3.1 Multimodal Image Registration

**Fig. 5:**
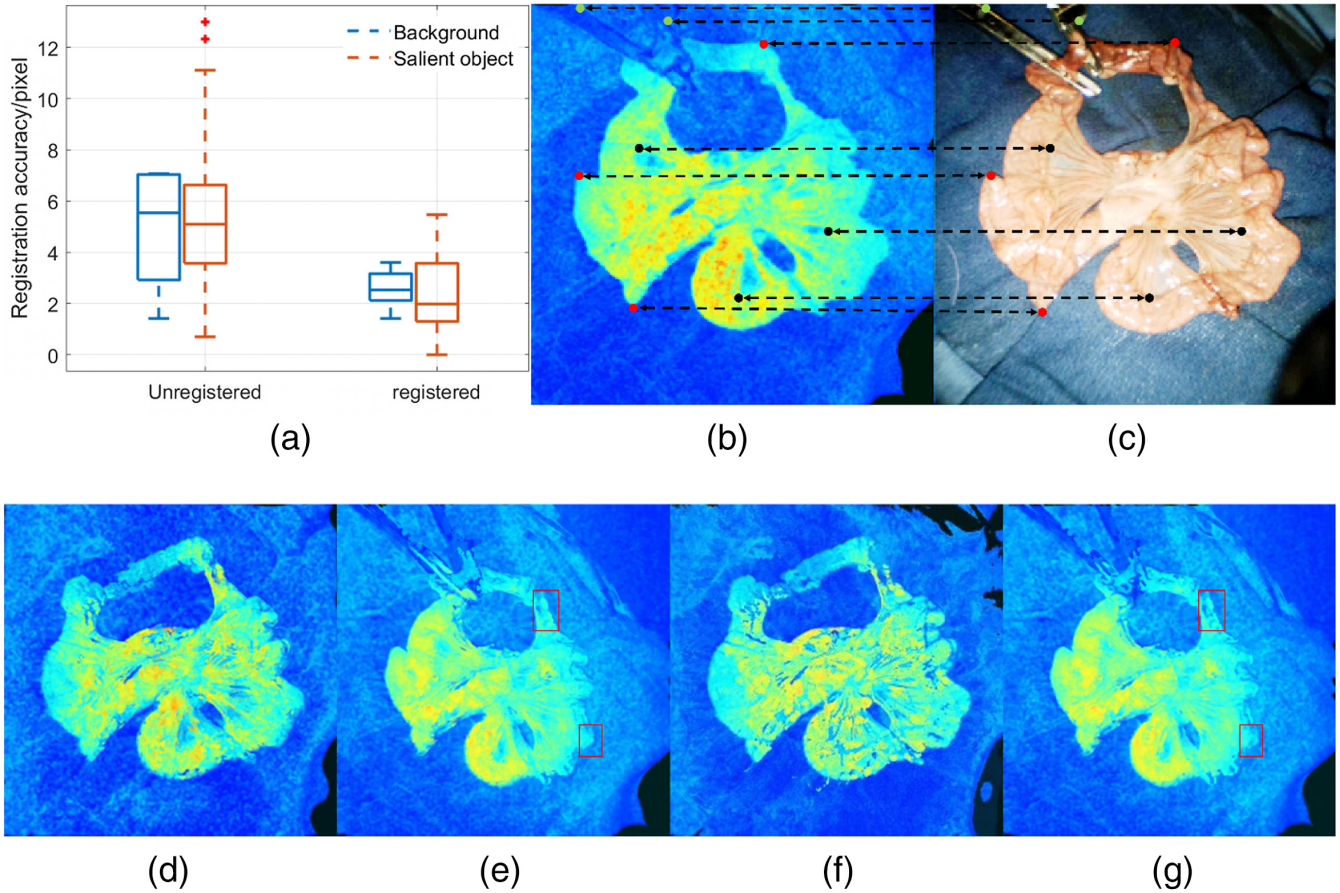
(a) Registration accuracy in pixels analyzed with and without using the proposed model; (b) and (c) annotation samples between RGB/LSCI images; (d) image registration results with $L_{\text{smooth}}$; (e) image registration results with the bilateral filter; (f) image registration results without $L_{\text{smooth}}$; and (g) image registration results without the bilateral filter.

**Fig. 6:**
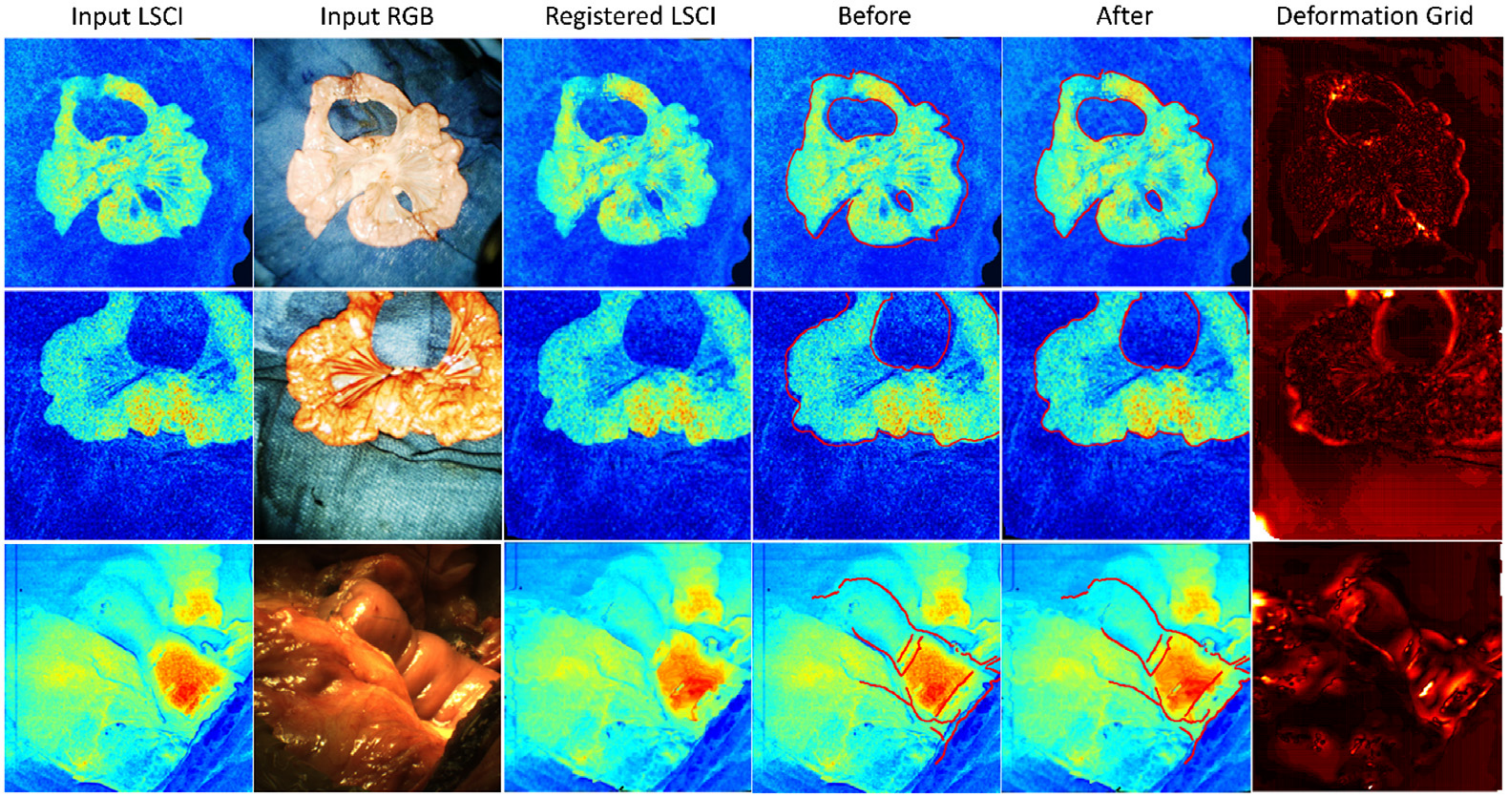
Image registration results between RGB/LSCI images.

The quantitative results shown in Fig. 5 verify the effectiveness of multimodal image alignment for salient peripheries, blurry vessels inside, and background. This shows that our model can preserve the geometric details and prevent unnatural deformations during image registration. Compared to the background landmarks in green, the red landmarks are more important because the edge alignment significantly affects the subsequent similarity measurement (Eq. 10). However, improving foreground accuracy should not be achieved at the expense of a large background distortion, which can mislead surgeons. Therefore, the merits of the two classes of landmarks were combined in measuring the Euclidean distance of the corresponding points to assess the performance of our model. Figure 5(a) shows the average distances between the target points with and without registration. The full scene had 256×256  pixels. Given a rough estimate of the first sample in Fig. 6, the width of the low-perfusion region was approximately 30 to 50 pixels, and the width of the right transition zone from abnormal to normal tissue was <10  pixels. In this case, if the registration step is bypassed, the overlapping of the ischemia probability maps and the original RGB images would be inaccurate, with errors around 5 pixels, as shown in Fig. 6. This order of magnitude is sufficiently large to identify an incorrect transitional necrotic region. The quantitative results in Fig. 5 verify the effectiveness of multimodal image alignment, both for salient and background objects. Furthermore, some remaining errors in the output images (generally <2  pixels) can be eliminated in the subsequent post-processing step on ischemia detection, where morphological operators are used to remove small bright dots. The image registration results using $L_{\text{smooth}}$ [Fig. 5(d)] and bilateral filtering [Fig. 5(e)] are also presented. Removing either of them could lead to discontinuous objects and rough edges, both locally and globally, as shown in Figs. 5(f)–5(g), highlighted by the red rectangles.

As shown in Fig. 6, our network can perform precise nonrigid image registration using different modalities. A Canny edge detector was used as a reference to define the boundary of the input RGB images, which was overlaid onto the input and registered LSCI images, as shown in the fourth and fifth columns. The sixth column represents the deformation grid from the LSCI to RGB images, which is a heatmap that combines the two-dimensional displacement vectors into brightness variations. As observed, large deformations occurred in the background or periphery of the tissue, which ensures that most part of the object remains unchanged. For example, in the first row of Fig. 6, the orange regions of the high-speed flow have similar patchy distributions before and after registration. In the second example in Fig. 6, the large deformation near the inside of the hole did not change the physical form of the mesenteric vessel. Furthermore, different intensity variations between LSCI and RGB near the corner of the frame may explain the large deformation fields on the lower left. However, this only applies in the background, as the bowel maintains well aligned. The third example shown in Fig. 6 shows a portion of the healthy small bowl inside the abdomen without a blue surgical towel. This interferes with the complex background and fuzzy edges. Unlike the second example, which has branching of a distinguishable superior mesenteric artery, limited mutual morphological features are observed between the LSCI and RGB data. Under these circumstances, the intestinal and abdominal walls still aligned well without interior distortion. This validated the robustness of our proposed model against a complicated surgical scene and limited mutual information. Our model works both ways, implying that a reverse spatial transformation is also practicable. However, our experiments verified that swapping the LSCI/RGB images performed significantly worse. The key reason is that standard RGB images contain more detailed morphological features that lack in speckle images, hindering the generation deformation grids of RGB images. Owing to bilinear interpolation, blurry areas with lengths of 0 to 3 pixels could occur in the registered LSCI images. This effect on ischemia detection is negligible because the fuzzy regions are considerably smaller than the main features extracted by the model, namely, the branching of the superior mesenteric artery, stitches, and intestinal walls. By contrast, the blurry areas mainly covered tiny capillaries in the LSCI images with little detail. These regions have low contrast, and the blurring caused by interpolation makes their brightness values more homogenous. This does not affect the similarity measurement stated in Eq. 10, where we focus on the overall difference between the speckle patches.

**Fig. 7:**
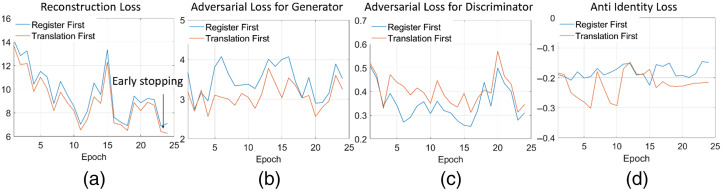
(a)–(d) Plots of the loss curves in image registration network.

In Fig. 7, the impact of different loss terms on the training procedure is explored. The reconstruction loss proved that the similarities between the output of LSCI and input RGB images increased during training. $D_{2}$ and $G_{2}$ were optimized through competition, as demonstrated by the wavy shape of the adversarial loss. Similar results from the R·T and T·R pipelines validate the orthogonality of the alignment and translation modules, which can be implemented independently without interference. Adding the $L_{\mathrm{anti}}$ term penalizes the zero-deformation grid, which encourages $R_{1}$ to explore the geometric details of the input data.

### 3.2 CGAN-based Ischemia Detection

**Fig. 8:**
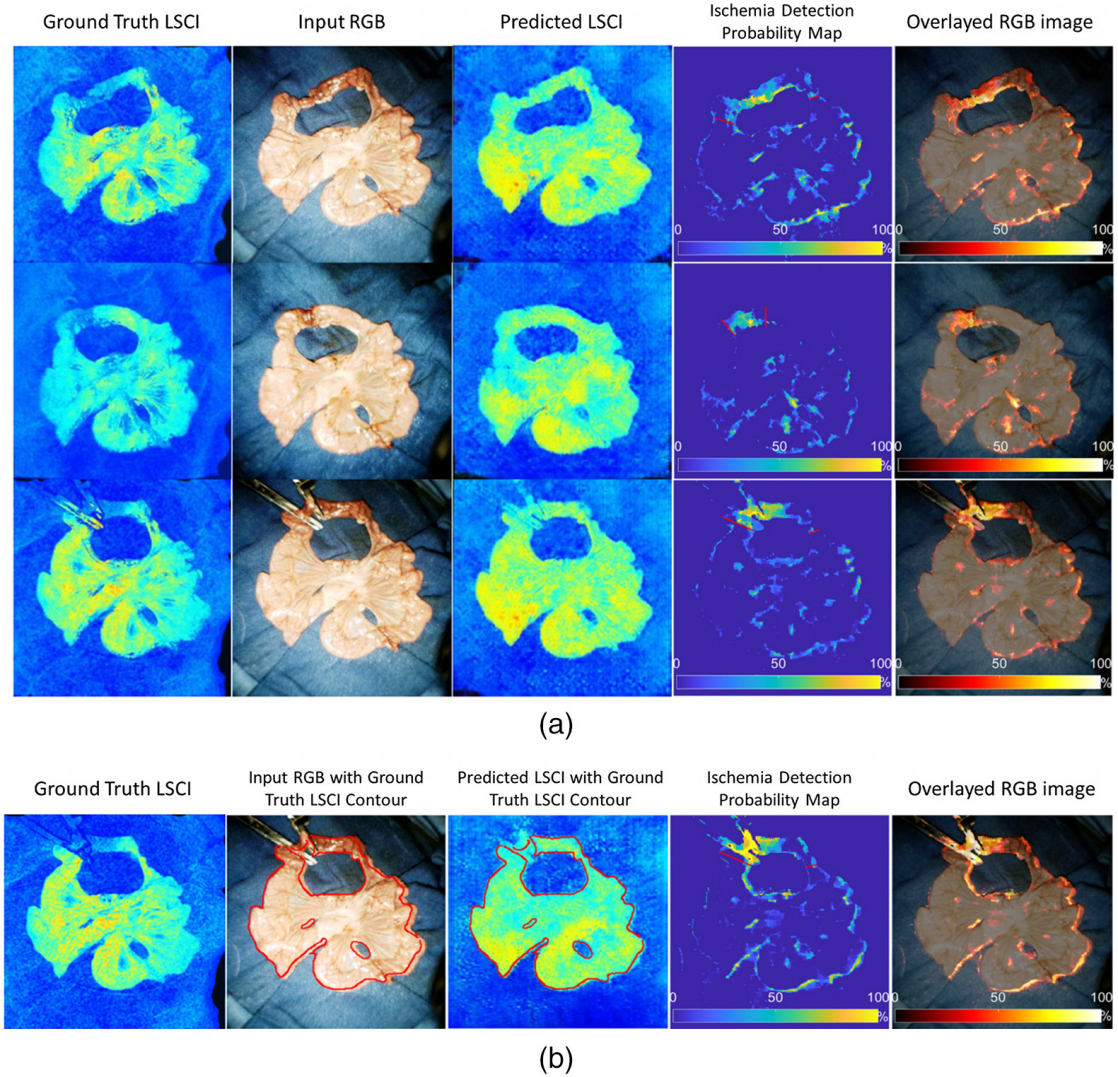
(a) and (b) Examples of the proposed ischemia detection network.

**Fig. 9:**
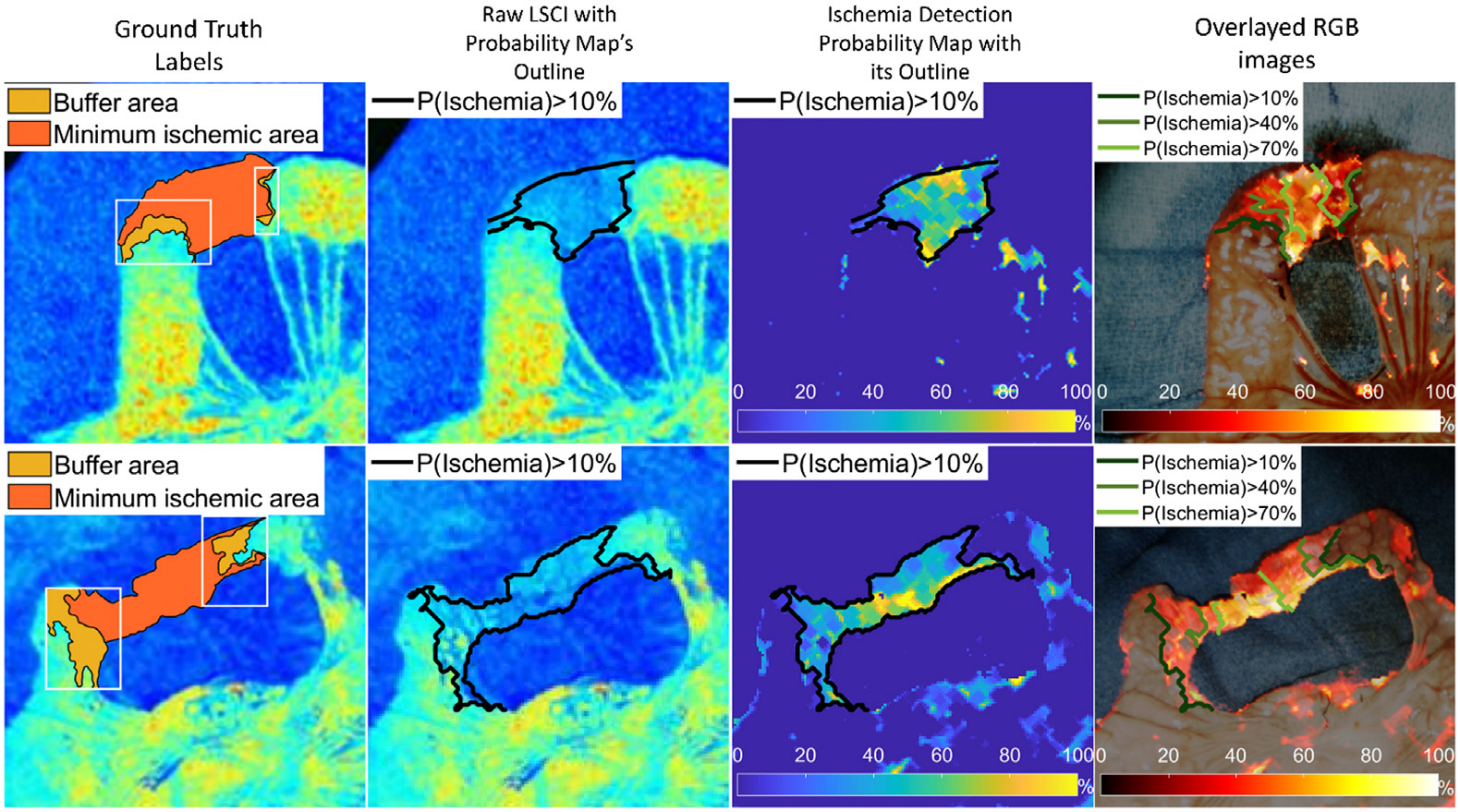
Enlarged examples of the proposed ischemia detection network. From left to right: ground truth labeling results by selecting the ROI (white box) and calculating the contour maps (black lines inside the box); downsampled LSCI images covered by the outline from the probability map when P(Ischemia)>10%; ischemia detection probability map covered by the outline when P(Ischemia)>10%; and overlayed high-resolution RGB images with the probability map and the predicted boundaries.

Figures 8 and 9 show a case of ischemia detection using our method. The resection margins labeled by the surgeon were marked in red. First, the strong generative power of our model was validated with RGB images as conditional features, as shown in the third column of Fig. 8. The representation of the target bowel was successfully transferred into the field of LSCI, generating realistic-looking speckle images even with a previously unseen pathological dataset. As described above, our network only learns the multimodal correspondence from the healthy tissue. When considering the abnormal region, only the spatial information is retained and there is a large deviation in the intensity distribution between the output and ground truth. This suggests that our model can properly reconstruct normal vascular images from the encoded latent space. Then, an anomaly score was defined in percentage form, as in Eq. 10, to compare the output of LSCI with the raw LSCI, as shown in the fourth column. Here, the output speckle images served as a “baseline,” demonstrating that LSCI did not require further calibration. The intensity level of traditional speckle images depends on the blood flow, shutter speed, polarization effect, etc..^13^ It indicates the relative velocity of blood flow and is thus inconvenient for a comparison between samples. By subtracting the output baseline from raw LSCI, these factors can be excluded because the normal RGB/LSCI correspondence has been already learned in training. Only the reconstruction error is highlighted in the pixelwise residual map, which is attributed to abnormal tissue perfusion. Based on the first and fourth columns, our model is sufficiently robust to distinguish normal and abnormal tissue under different conditions by comparing it with surgeons’ labels covering larger regions containing parts of normal tissue perfusion patterns. Registration errors of 0 to 2 pixels explain some false alarms of bowel ischemia on the object outlines. However, in most cases, they appear as dots with a lower intensity, far from the target ischemic areas and can be easily identified by surgeons. The probability map was further overlaid on the standard RGB images in the fifth column, and both are enlarged in Fig. 9.

In Fig. 9, the second type of ground truth is utilized. The dominating intensity levels of contour lines vary for different bowel patterns, making it difficult to set a unified critical value for ischemic segmentation. Using adjustable thresholds here could increase the generality of manual annotations. According to the contour lines, the minimum ischemic area was generated outside the buffered area, where both the normal and abnormal labels were considered correct, based on the second ground truth type shown in Fig. 9. The buffer area can be considered a more general and wider boundary that is closer to the ideal values. This reduced the uncertainty in identifying intestinal ischemia; thus, our model could be effectively evaluated without inaccessible ideal labels. The averaged contour lines of LSCI images are consistent with our probability map, which proves that our model can categorize healthy and pathological tissues similarly as LSCI images. It is worth noting that the original high-resolution images (1024×1024  pixels) are employed in the fourth column and the probability map is resized by four. The results show an acceptable alignment between the high-resolution color images and the up-sampled probability map. Some reconstruction errors are observed on the mesentery, which has a delicate structure with a large number of blood vessels. Another type of error is due to the grainy nature of speckle images, resulting in spots of normal labels inside the abnormal regions in the probability map. However, these errors do not affect the overall partitioning of intestinal ischemia. Based on Figs. 8 and 9, our model still displays strong capabilities, helping surgeons identify resection areas and make intraoperative decisions in intestinal anastomosis.

To further explore the impact of the registration step as an upstream task, bowel ischemia detection was implemented without image registration, as shown in Fig. 8(b). Compared to the input images, the output positions of the bowel were changed and closer to the ground truth. In this case, the cGAN-based model simultaneously learned RGB/LSCI photometric mapping and spatial transformation. However, it could not achieve precise alignment, leading to more errors near the edge. In contrast to Fig. 8(a), the highlighted pattern shrunk on the left side. Based on the surgeons’ annotations and the original LSCI images, the left transition zone was incorrectly estimated. The results show that the image registration step is necessary to correctly identify the boundary between the healthy and pathological areas.

The corresponding quantitative evaluation results are given in Table 2. We employed the second type of ground truth described above, which consists of the averaged contour lines in the transition zones. The field of view was narrowed to ∼80×100  pixels for each image. This can prevent class imbalance and render the accuracy matrix more representative. A large AUC (0.9305) validated the strong capability of our model in detecting intestinal ischemia. If the registration step is bypassed, a significant decline in performance is observed with the same setup. This emphasizes the requirement of registration, as it eliminates pixel-wise misalignment errors in the anomaly score r and thus increases the model accuracy. Finally, our model achieved an accuracy of 93.06% and a specificity of 94.83%. Measuring the similarity of positive pixels between the ground truth and predicted images and excluding the true negative pixels resulted in a Dice coefficient of 90.77%, which is indicative of good performance.

**Table 2:** Evaluation result of RGB/LSCI dataset.

	Accuracy	Dice coefficient	Sensitivity	Specificity	Precision
With registration	93.06%	90.77%	89.34%	94.83%	92.77%
W/o registration	88.34%	84.04%	81.01%	92.26%	87.91%

## 4 Discussion

In this study, a novel computer-assisted optical platform, a cGAN-based unsupervised deep learning method, was proposed to detect ischemic regions and assess tissue perfusion levels in bowel. Quantitative detection of these abnormalities can complement the surgeon’s subjective judgement through visual tests. Specifically, a GAN, combined with conditional features of color RGB images, was designed to predict a healthy vascular flow map and identify pathologic regions by calculating the residuals using ground truth LSCI images. An unsupervised method is important for detecting bowel ischemia because the ground truth annotations are subjective and unavailable. Because of theoretical limitations, the relative tissue perfusion levels provided by LSCI do not display any significant correspondence with the actual necrosis rate.^18^ Thus, inexperienced surgeons do not take full advantage of this when assessing intestinal viability. Another obstacle is the morphologic variability of bowel ischemia, which are dependent on pathophysiology, severity, duration, etc.^43^ Our unsupervised model does not limit to a single morphological feature set, and any differences from the learned healthy norm can be detected. In this context, some algorithms of AEs and their advanced versions have been reported to be useful in MRI anomaly segmentation along with the same ideas presented above.^22^^,^^25^ In this study, the cGAN was considered to be an optimal model for detecting the ischemic intestine. Compared to AEs, GAN is not restricted to the bottleneck z-space and tends to have a stronger ability to generate realistic new samples without blurring.^44^ Governed by the dimensions of our dataset, one-channel data were used as conditional features, providing additional prior knowledge during training. However, one potential drawback of this relatively complicated design is overfitting. As described above, various experimental constraints were set to increase data diversity. A 50% dropout rate was set to reduce the overreliance on layers. Our results indicate that the cGAN-based model can clearly differentiate abnormal tissue perfusion regions from healthy ones in RGB/LSCI data with an accuracy of 93.06% and a Dice coefficient of 90.77%. The high accuracy demonstrated its strong ability to predict based on unseen data that share little mutual information with training groups. To the best of our knowledge, this is the first study using cGAN for unsupervised anomaly detection in diagnosing tasks in medical imaging, particularly using a combination of visible light and speckle imaging.

Our findings also demonstrate the utility of the combined cGAN and STN models for multimodal image alignment, which can reduce the errors in the subsequent multi-source information fusion task. One of the most challenging data groups to assess would be intra-abdominal images, which lack a clear background. Thus, it is quite difficult to define a suitable metric to measure cross-modality similarity; some existing ones^45^^,^^46^ are not sufficiently robust for our dataset. Some conventional methods also struggle with extracting and matching features owing to limited geometric details. In our model, these metrics were bypassed and a simple mono-modality measurement was employed. In addition to the common loss functions used in cGAN, objective $L_{\text{smooth}}$ and bilateral filters were introduced to prevent unnatural image distortion. The resulting spatial transformation generated by our model was continuous and global. Another objective function was further designed based on the original network architecture^31^ to prevent convergence to a local minimum rather than a global optimal solution. Moreover, it is an unsupervised model that does not require perfectly aligned ground truth data, which do not exist in most practical clinical settings. Considering its performance on complex abdominal scenes, the modified model has potential for being widespread in medical image registration tasks,^47^ such as laparoscopic surgery.

This study has also revealed some limitations that that need to be addressed in future work. Considering the non-invasive real-time LSCI as the ground truth to assess the tissue perfusion level, further experiments that include further surgical interventions, such as bowel resection and anastomosis, can be conducted. Re-establishment of intestinal microcirculation and postoperative outcomes in animal subjects can be another reliable measurement for the optimal resection region. Moreover, some spotty or contiguous areas with false-positive results can be reduced. This is because of the limited capabilities of our model in reconstructing the mesentery area. Conducting additional animal studies can help increase the diversity of data. The learning procedures of our model can be optimized by varying the anatomical characteristics of the mesentery. Finally, the results reported in certain recent studies could be applied to our proposed method to further improve the stability of GAN, for example, Wasserstein loss for mode collapse^40^ and Monte Carlo dropout for stable outputs.^48^ Future work will also include online processing and more statistical tests on our network.

## 5 Conclusion

In this study, a computer-aided detection platform is proposed, combined with an unsupervised learning model of cGAN, to achieve a quantitative and objective evaluation of tissue perfusion levels. A dual-modality benchtop imaging system was employed to collect standard RGB and LSCI images of intestinal tissues in preclinical swine studies. Two different cGAN extensions were employed for multimodal image-to-image registration and translation. Our model could predict healthy tissue perfusion patterns from color RGB images, thereby recognizing ischemic areas at risk, with an accuracy of 93.06% and a specificity of 94.83%. The model showed an accurate assessment of pixelwise probability distribution of intestinal ischemia, outperforming the raw LSCI images. This shows that LSCI does not require further calibration with a sample-dependent baseline, being more convenient for interpatient comparisons. Furthermore, the proposed model is more capable of indicating ischemic or necrotic bowel tissue and provides a clear and accurate segmentation between normal and abnormal tissues for surgical intervention. In particular, the method can help surgeons with intraoperative diagnosis and treatment settings for acute mesenteric ischemia and intestinal anastomosis.
